# Development of an Ex Vivo Platform to Model Urethral Healing †

**DOI:** 10.3390/mps8040096

**Published:** 2025-08-15

**Authors:** Christopher Foster, Ryan Tran, Khushi Grover, Abdullah Salama, Courtney K. Rowe

**Affiliations:** 1 Department of Pediatrics, University of Connecticut School of Medicine, Farmington, CT 06032, USA; 2 College of Osteopathic Medicine, University of New England, Biddeford, ME 04005, USA; 3 Division of Pediatric Urology, Connecticut Children’s, Hartford, CT 06108, USA

**Keywords:** urethra, wound healing, growth factor, regenerative medicine

## Abstract

Background: Urethral strictures impact millions, causing significant morbidity and millions in healthcare costs. Testing new interventions is limited by the lack of inexpensive urethral healing models. We developed an ex vivo model of early urethral wound healing using explanted rabbit urethral tissue. This was used to test the impact of six growth factors (GFs). Methods: The rabbit urethra was detubularized by cutting it between the corpora cavernosa, and then it was stitched flat using a custom 3D-printed platform. The tissue was carefully scratched to produce a visible wound, and the specimens were placed in media containing growth factors at 100 ng/mL and 10 ng/mL. Images were taken at 0, 24, 48, 72, and 96 h, and the wound area was measured by blinded reviewers to determine the rate of wound contraction. Results: Specimens with IGF at 100 ng/mL showed a statistically significant difference in wound contraction when compared to those with GF-free control medium, showing that IGF-1 supports early urethral epithelization and may improve healing. Conclusions: The developed protocol provides a simple explant platform that can be used to investigate methods of enhancing early phases of urethral healing or used to investigate other areas of urethral health, including drug delivery, infection, and mechanical properties.

## 1. Introduction

Urethral strictures account for almost 1.5 million yearly office visits, totaling almost USD 200 million in healthcare costs [1]. Strictures most commonly result from idiopathic causes (41%) and iatrogenic causes (32–45.5%), and they significantly impact the quality of life for patients [2,3,4]. An intervention to promote postoperative healing following common procedures, such as transurethral resections and hypospadias repairs [2], would be of significant benefit.

The urethra re-epithelializes after injury or surgery via ingrowth from the edges of the defect, similar to that of re-epithelialization of the dermis [5,6,7]. Early epithelialization of the urethra appears to be the key difference between healthy healing and the fibrosis that causes urethral strictures [8]. It is theorized that the faster the urothelium closes, the better for healing, as urinary extravasation into surrounding tissue increases the inflammation surrounding the wound and the risk of fibrosis with subsequent urethral stricture [9]. However, the mechanism currently thought to be the driver of urethral re-epithelialization appears to be this very exposure to urine, which causes proliferation and migration of surrounding cells in addition to the fibrosis-causing inflammation [10]. Ultimately, what is needed is a way to increase epithelialization without causing inflammation, which is anticipated to reduce stricture and complications after urethral injury or surgery.

Research in this area has been limited due to challenges in finding an appropriate model to emulate urethral healing in vitro, which prevents further investigation into possible treatments to enhance effective healing. While living animals could be used to discover new interventions for healing, the resources and maintenance required for these in vivo studies are intensive, and the ethical goal is always to reduce the number of research animals needed. Current in vitro urethral healing models often use urothelial cells from the bladder because urethral cells are not easily cultured and are not commercially available [11,12,13,14,15,16,17]. A previously developed 2D wound healing assay by our group used human urothelial cells with various growth factors (GFs) [18] and showed similar stages of urethral healing to the in vivo studies using animals [5,6,7]. However, the relevance of this 2D model to in vivo healing may be limited because urothelial cells of bladder origin have different embryologic origins and different in vitro growth potential when compared to urothelial cells of urethral origin [19]. An alternative platform that uses the entire urethra would allow for in vitro research that is more clinically relevant, and would provide a more rapid translation to live animal research and eventual clinical practice.

The goal of this project was to develop an ex vivo platform for the three-dimensional modeling of early urethral healing using explanted rabbit urethra and to evaluate the differences in model wound contraction between different GFs. The protocol was based on prior research using explanted penile tissue [20] and modified over the course of two years. The rabbit urethra was selected as the basis of this model, as rabbits are the most commonly used live animal model to study urethral injury or surgery [21,22,23,24]. Using the same species for in vitro work allows the findings from this 3D protocol to be rapidly translated to in vivo animal research. The technique described to detubularize and maintain the urethra has applicability beyond the study of early healing, and could also be used to evaluate new drugs, urethral infections, or for mechanical testing of the urethra.

## 2. Materials and Methods

### 2.1. Materials for Model Creation

1. Phosphate-buffered saline (PBS; Gibco, Waltham, MA, USA; ThermoFisher Cat. no.: 10010023).
2. Penicillin–Streptomycin (Corning, Glendale, AZ, USA; Fisher Scientific Cat. no.: MT30001CI).
3. Dulbecco’s Modified Eagle Medium (DMEM; Gibco, Waltham, MA, USA; ThermoFisher Cat. no.: 11965118).
4. Fetal Bovine Serum (FBS) (Corning, Glendale, AZ, USA; Fisher Scientific Cat. no.: MT35016CV).

### 2.2. Equipment for Model Creation

1. Large sharp scissors, forceps, small sharp scissors, and a needle driver.
2. Disposable centrifuge tubes with a 15mL flat cap (Fisherbrand, Waltham, MA, USA; Fisher Scientific Cat. no.: 07-200-886).
3. An original Prusa i3 3D printer (Prusa Research, Prague, Czech Republic).
4. Prusament PLA Pristine White, 1 kg (Prusa Research, Prague, Czech Republic).
5. PDS II Suture, Size 4-0, SH, 27” (Ethicon, Cincinnati, OH, USA; Fisher Scientific Cat. no.: 50-118-0707).
6. #15 protected disposable scalpel stainless steel blade (Bard-Parker, Caledonia, MI, USA; Fisher Scientific Cat. No.: 02-688-80).

### 2.3. Materials for Growth Factor Testing

1. Prostate Epithelial Basal Medium (ATCC, Manassas, VA, USA; ATCC Cat. no.: PCS-440-030).
2. Corneal Epithelial Cell Growth Kit (ATCC, Manassas, VA, USA; ATCC Cat. No.: PCS-700-040).
3. Recombinant Human EGF (PreproTech, Cranbury, NJ, USA; ThermoFisher Cat. No.: AF-100-15-500UG).
4. Recombinant Human FGF-basic (PreproTech, Cranbury, NJ, USA; ThermoFisher Cat. No.: AF-100-18B-50UG).
5. Recombinant Human IGF-1 (PreproTech, Cranbury, NJ, USA; ThermoFisher Cat. No.: AF-100-11-500UG).
6. Recombinant Human PDGF-BB (PreproTech, Cranbury, NJ, USA; ThermoFisher Cat. No.: AF-100-14B-10UG).
7. Recombinant Human TGF- β1 (PreproTech, Cranbury, NJ, USA; ThermoFisher Cat. No.: AF-100-21C-10UG).
8. Recombinant Human VEGF (PreproTech, Cranbury, NJ, USA; ATCC Scientific Cat. No.: AF-100-20-10UG).
9. Calcein AM (Invitrogen, Waltham, MA, USA; ThermoFisher Cat. No.: C3100MP
10. Ethidium Homodimer I (Biotium, Fremont, CA, USA; Fisher Scientific Cat. No. 50-196-4627).

### 2.4. Specimen Collection

**Figure 1:**
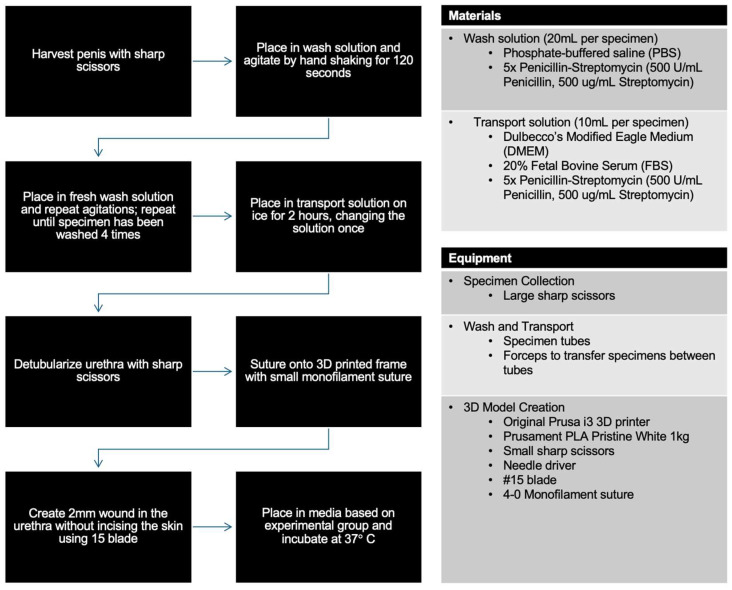
Model creation workflow, materials list for each solution, and equipment list for each step.

Rabbit penises from male New Zealand White and Silver Fox breeds were harvested at 12–14 weeks of age (Figure 1). The selection of the breed was based on the availability from our specimen supplier. The entire penis and urethra were harvested immediately after animal sacrifice using large, sharp scissors. It is imperative to minimize the hair-bearing skin taken along with the specimen, as hair is thought to contribute to bacterial contamination.

IACUC approval was not required, as the tissue was harvested after sacrifice from animals that were used for other purposes. To reduce any variables while developing this model, we elected to use specimens from animals that had neither undergone any prior procedures nor received any prior medication. But for future work, animals who received procedures or medications that do not impact overall healing or the urinary tract would also be a good resource for specimens. Additionally, we anticipate that any rabbit breed could be used for this model. There is no known difference in the urethral stricture rates based on race in humans, and so we do not expect a difference in model outcomes based on rabbit breed [25].

### 2.5. Wash and Transport

Wash solution was prepared using phosphate-buffered saline (PBS; Gibco, Waltham, MA) and 5× Penicillin–Streptomycin (Corning, Glendale, AZ, USA). Transport solution was prepared using Dulbecco’s Modified Eagle Medium (DMEM; Gibco, Waltham, MA, USA), 20% Fetal Bovine Serum (FBS; Corning, Glendale, AZ, USA), and 5× Penicillin–Streptomycin. 20% FBS refers to the concentration of FBS within the medium, and 5× Penicillin–Streptomycin refers to a concentration that is five times stronger than the typical working concentration (ie, 500 U/mL Penicillin, 500 ug/mL Streptomycin).

Whole specimens were placed in a 15 mL disposable centrifuge tube (Fisherbrand, Waltham, MA, USA) containing 5 mL of wash solution, and were then agitated by shaking by hand for approximately 120 s. The specimens were then removed and placed in a separate container of wash solution for another round of agitation. Four rounds of washing and agitation were performed per specimen. The specimens were then placed in 5 mL of transport medium and placed on ice. They were left to soak for a total of 120 min, changing the medium once during that time period.

Our main issue with the transport of specimens was bacterial contamination, which affected approximately 20% of the early collections. The above protocol was developed and modified to reduce contamination and has eliminated this issue to date.

### 2.6. 3D Model and Wound Creation

**Figure 2:**
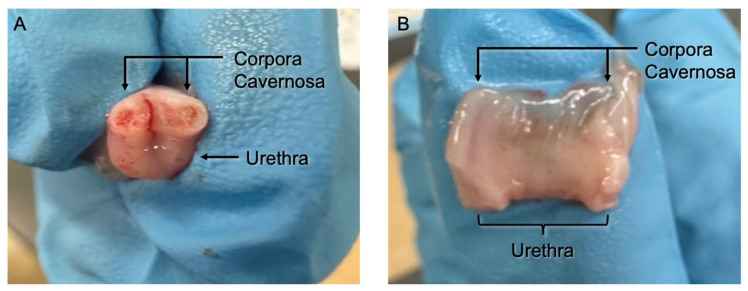
Rabbit penis with an initial cut through the urethra between the two corpora cavernosa (**A**). Urethra detubularized (**B**). Adapted from Tran et. al. [26] with permission from Wolters Kluwer Helath, Inc, 2025.

Rabbit penises were detubularized to expose the urethral surface by cutting them with small sharp scissors between the two corpora cavernosa (Figure 2A) until the urethra was fully detubularized and flat (Figure 2B). Placing one of the scissors’ tines within the lumen of the urethra ensures that this step is performed correctly. We have not found it as crucial to perform this step in a sterile fashion as it is to perform the above washing and transport protocol.

**Figure 3:**
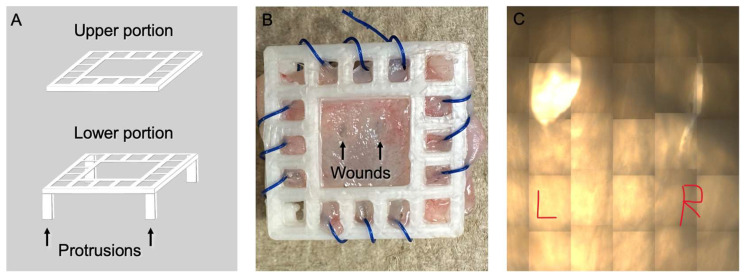
Two pieces of the 3D-printed platform. The tissue was placed between the upper and lower portions, then rotated 180 degrees to be placed in the media. Protrusions in the lower portion hold the tissue in the media and prevent floating (**A**). Detubularized tissue sutured within the platform (**B**). Microscopic images with left (L) and right (R) wounds identified for reviewers (**C**).

A custom 3D platform was designed and printed (Figure 3A) using the Original Prusa i3 3D printer (Prusa Research, Prague, Czech Republic). The filament used was the Prusament PLA Pristine White, 1 kg (Prusa Research, Prague, Czech Republic). The code for printing is available on request from the authors. Platforms have a base and a cover section, along with openings for sutures. The platform was designed in an upper and lower portion to distribute tension across the specimen while securing the urethra and preventing tissue contraction or tearing. Because the PLA filament floated in media, this design included raised protrusions to keep the specimen within the media. The authors anticipate that any 3D printer and filament could be used for this purpose. The platforms were sterilized by spraying them with Ethanol and placing them in a cell culture hood under UV light for 30–120 min before use. As the platforms were fragile, they were only used once.

The urethras were secured between the two sections of the 3D platform using a monofilament suture (Figure 3B); in this example, 4-0 PDS (Ethicon, Raritan, NJ, USA). A #15 blade (Bard-Parker, Caledonia, MI, USA) was used to create wounds that were approximately 2 mm in length on the surface. We found two wounds to be ideal per specimen, as more wounds caused the specimen to tear. The wounds were made large enough to be visualized with the naked eye so that they were easily visible with low magnification microscopy. The wound size could not be standardized, which was accounted for in the data analysis. Care was taken for the wound to include only the thin urothelial surface and not the layer of skin behind it, which was facilitated by ensuring that the wound was between the two corpora, which is where the most structure is, and the model is held most securely. Preventing skin damage is essential to provide a physiologic model of urethral injury, and this is why a punch was not able to be used to create the wounds.

### 2.7. Controls and Evaluated GFs

Two controls were selected based on prior published use for urethral modeling. [18] The GF-containing medium describes the combination of commercially available Prostate Epithelial Basal Medium (ATCC, Manassas, VA, USA) and Corneal Epithelial Cell Growth Kit (ATCC, Manassas, VA, USA), which are sold by the American Type Culture Collection so as to be used together for cell growth. The Corneal Epithelial Cell Growth Kit contains EGF, TGF-α, KGF, and Extract P. This was considered a positive control that best mimicked the physiological conditions. We also used the GF-free medium (Prostate Epithelial Basal Medium; ATCC, Manassas, VA, USA) as a control that did not contain any GFs. This was considered a negative control.

Six candidate GFs (PreproTech, Cranbury, NJ, USA) were selected based on their role in urethral or wound healing [18]: epidermal growth factor (EGF), fibroblast growth factor-basic (FGF-basic), insulin-like growth factor-1 (IGF-1), platelet-derived growth factor (PDGF), transforming growth factor beta (TGF-β1), and vascular endothelial growth factor (VEGF). The urethras were placed in Prostate Epithelial Basal Medium (i.e., GF-free medium) with the addition of the specified GF at 100 ng/mL and 10 ng/mL.

The samples were place in the media, as above, and incubated at 37 °C.

### 2.8. Imaging and Analysis

The urethras were imaged using the Zeiss Observer Z1 (Zeiss Group, Oberkochen, Germany) microscope at 5× magnification at 0, 24, 48, 72, and 96 h. The time frames were selected to mimic the timeframe of in vivo urothelial healing after urethral surgery or injury [5,6,7]. The entire specimen was imaged, and a composite was created by tiling the images using the included Zeiss software (Figure 3C). The images that were saved included both scratches and the 3D frame, which was 28 tiles (7 across and 4 down). The Zen Blue software auto calculates the tiling and overlap with a default 10% overlap. The images were blinded using a random number for the images and for the timeframes. The blinded images were evaluated by 3–4 reviewers, depending on the reviewer’s availability (R.T., K.G., A.S., and C.K.R.). The reviewers drew the outline around the wound in Image-J software Version 1.53 [27], which was then used to calculate the area. We elected to use blinded reviewers as there was too much variability in the wound sizes and images for Image-J to automatically detect the edges of the wound.

The results were normalized to the percent of the initial wound area at time 0, with the negative numbers showing a decrease in the wound size, and the progressively decreasing percentages indicating wound contraction. We elected to normalize to time 0, given the inability to standardize the wound sizes. We did not attempt to resolve disagreements between reviewers; instead, we elected to calculate the mean wound area as a percent of the initial wound area for each time point. This allowed for the measurements to account for the differences in wound size between specimens as well as the differences in reviewer perception of the wound edges.

Descriptive and comparative statistics of the wound area were performed in Excel. Descriptive statistics were performed on both the unnormalized measurements and the wound area that was normalized as a % of time 0. This was performed for each wound (four per GF and concentration, with 3–4 reviewers for each wound) at each time point and concentration. The comparative statistics included two-factor ANOVA and linear regression. A pairwise two-factor ANOVA with replication was performed using the mean of the reviewers’ measurements of the wound area (as a % of time 0) for each wound at each time point and concentration. Each GF and the positive control were compared in a pairwise fashion to the GF-free media (negative control). For each concentration and both controls, linear regression was performed, and the regression lines plotted. These were used to evaluate that the direction of significance in comparison to the negative control was in the direction of the positive control. Statistical significance was set at *p* < 0.05.

### 2.9. Cell Viability

At 96 h, the specimens were washed with PBS, incubated with Calcein AM (Invitrogen, Waltham, MA, USA) and Ethidium Homodimer (Biotium, Fremont, CA, USA) stain for 15 min, and then evaluated using the Zeiss Observer Z1 (Zeiss Group, Oberkochen, Germany) microscope at 10×. Our lab uses the Invitrogen Live/Dead protocol described, which is part of the product information for their LIVE/DEAD ^®^ Viability/Cytotoxicity Kit (Invitrogen, Waltham, MA, USA; ThermoFisher Cat. No.: L3224), though we no longer purchase the kits and prefer to purchase supplies separately.

Of note, we were unable to image over 10× magnification due to the thickness of the tissue, which resulted in dark images at high magnification. Because of the variations in tissue height, it was also difficult to obtain high-resolution images at 10×, as the areas of each image were often out of focus. Because of these limitations, we felt that cell counting would be inaccurate and, instead, we elected to perform a subjective evaluation of the calcein AM and ethidium homodimer fluorescence.

## 3. Results

### 3.1. 3D Wound Model

**Figure 4:**
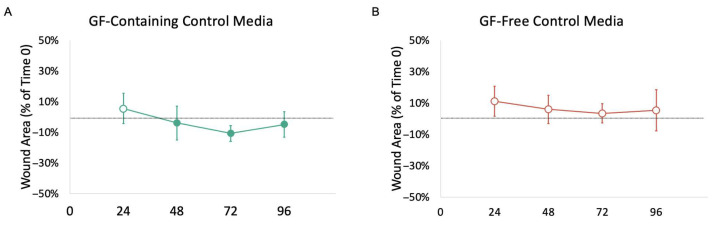
Mean wound area as a percent of time 0 over time for GF-containing (**A**) and GF-free (**B**) control media. Negative values indicate a decrease in wound area from time 0 and are indicated by a solid circle. Positive values indicate an increase in wound area from time 0 and are indicated by an open circle. GF-containing medium showed a statistically significant difference in mean wound area as a percent of time 0 (*p* = 0.0056) using pairwise two-factor ANOVA with replication.

The GF-containing medium showed a statistically significant difference in the mean wound area as a percent of time 0 (*p* = 0.0056) (Figure 4). The graph of the linear regression (not shown) showed a negative slope for both the GF-containing and GF-free media, indicating contraction of the wound over 96 h. The unnormalized values are provided in the Supplementary Information.

### 3.2. Impact of Growth Factors

**Figure 5:**
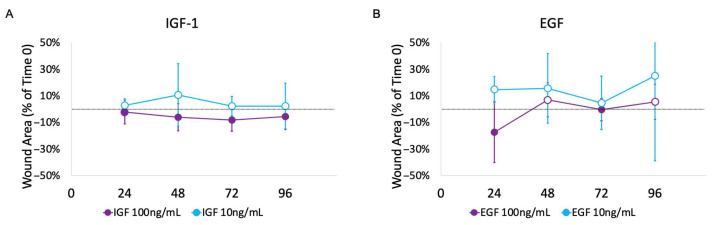
Mean wound area as a percent of time 0 over time for IGF-1 (**A**) and EGF (**B**) at concentrations of 100 ng/mL and 10 ng/mL. Negative values indicate a decrease in wound area from time 0 and are indicated by a solid circle. Positive values indicate an increase in wound area from time 0 and are indicated by an open circle. IGF-1 at 100 ng/mL showed a statistically significant difference when compared to the GF-free control medium (*p* = 0.0015) using pairwise two-factor ANOVA with replication.

IGF-1 at 100 ng/mL showed a statistically significant difference when compared to the GF-free control medium (*p* = 0.0015) (Figure 5A). The graph of the linear regression (not shown) showed a negative slope, indicating contraction of the wound. IGF-1 at 10 ng/mL had only positive wound areas with no statistically significant differences in the wound areas when compared to the GF-free control medium, though the graph of the linear regression was negative. Neither concentration of the EGF shows a statistically significant difference in the wound area when compared to the GF-free control medium. EGF at 100 ng/mL had a notable decrease in mean in the wound area at 24 h, followed by persistent increases (Figure 5B). This resulted in an overall positive slope of the linear regression, but without evidence of persistent wound contraction. All values of EGF at 10 ng/mL were positive, indicating a lack of any wound contraction.

**Figure 6:**
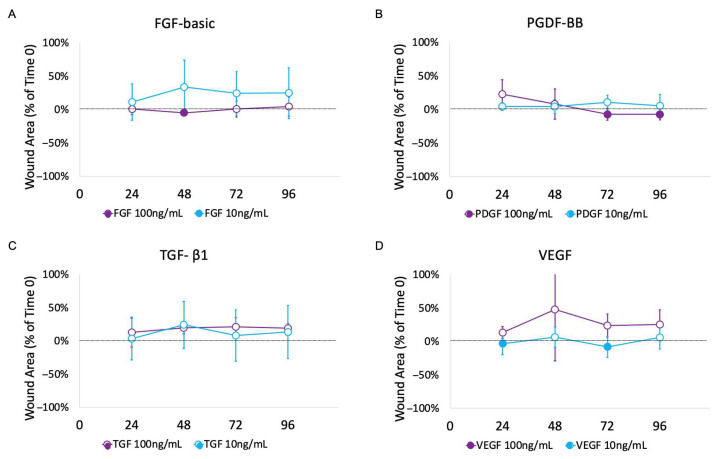
Mean wound area as a percent of time 0 over time for FGF-basic (**A**), PDGF-BB (**B**), TGF-β1 (**C**), and VEGF (**D**) at concentrations of 100 ng/mL and 10 ng/mL. Negative values indicate a decrease in wound area from time 0 and are indicated by a solid circle. Positive values indicate an increase in wound area from time 0 and are indicated by an open circle. TGF at 100 ng/mL showed a statistically significant difference when compared to the GF-free control medium (*p* = 0.0130) using pairwise two-factor ANOVA with replication, but in the opposite direction of the positive control. All measurements were positive, indicating a wound that increased in size after time 0.

There was no statistically significant difference between FGF-basic or VEGF at 100 ng/mL or 10 ng/mL and the GF-free control (Figure 6A,D). PDGF-BB at 100 ng/mL had negative values, indicating wound contraction at 72 and 96 h, but this was not statistically significant when compared to the GF-free control (Figure 6B). TGF- β1 at 100 ng/mL had mean wound areas that were significantly different from the GF-free controls, but all values were positive, showing a lack of wound contraction (Figure 6B,C).

### 3.3. Cell Viability

Cell viability on the specimens showed the presence of bright green calcein AM fluorescence with minimal red fluorescence of ethidium homodimer, indicating the presence of live cells within the specimen at 96 h. This was found in all tested specimens, including those GFs that showed no significant wound contraction (Supplementary Information).

## 4. Discussion

This study successfully developed an ex vivo 3D model of urethral wound healing by using explanted rabbit urethral tissue, with the ability to distinguish differences in wound contraction between different GFs. This explant model is inexpensive and simple for investigators to replicate using only tools that are commonly found in research settings. This new model allows for more complex studies of the urethra while minimizing research on live animals.

### 4.1. Benefit of This Ex Vivo Model over Other In Vitro Urethra Models

The native urothelium is a highly complex structure. The thickness of the endothelial layer varies depending on the distance from the bladder, and it contains mucus-secreting cells and solitary lymphocytes in addition to squamous or columnar epithelium [28]. A well-vascularized layer of connective tissue with smooth muscle running through it lines the epithelium and is called the corpora spongiosum in humans [28,29]. Around this is a surrounding layer of longitudinal and circular smooth muscle [30,31]. Attempts have been made to create de novo models of the urethra for in vitro studies, but given its complexity, their applicability to clinical use is limited. Versteeg et al. used primary urothelial cells on a collagen-fibrin hydrogel that was populated with fibroblasts to create what they call an organotypic reconstructed urethra [32]. Comparative histology shows that the organotypic urethra is significantly more uniform than the native urethral tissue, and only one urethral layer is replicated, with no spongiosal or muscular layers. Our 3D model provides the cellular complexity of a native urethra and includes both the spongiosal and muscosal layers as well as the surrounding skin. Casademont-Roca et al. expanded on Versteeg’s approach by developing a technique to add a layer of microvessel-like structures that are similar to the corpus spongiosum to develop what they call a “urethra-on-a-chip” [33]. However, this technique also fails to mimic the complexity and variability of either the endothelial or spongy urethral layers and lacks a smooth muscle layer. Our 3D model includes the actual spongiosum, as mentioned above, without the need for a substitute. In addition, both Versteeg and Casademont-Roca used cultured cells for their models. Culturing is known to alter the phenotype of urothelial cells, with an unclear impact on their behavior and response within an experiment [34]. While these investigators have shown great progress, their results still lack the complexity of the mammalian urethra. Because our 3D ex vivo model uses native rabbit urethral tissue, it contains the complex ecosystem of cells and connective tissue that make up the urethra, and therefore provides a more clinically relevant environment for in vitro testing.

While our ex vivo model is more complex than prior published in vitro models, it is less complex than the in vivo urethra. However, it provides a useful stepping stone for researchers prior to live animal work. This model is significantly cheaper than rabbit survival surgery. Specimens were obtained from our supplier for USD 30. The current price for a 2.5–2.8 kg New Zealand White Rabbit is USD 382 (Charles River Laboratories, Wilmington, MA, USA). Performing live animal research for urethral healing also includes the cost of housing animals before and after surgery, as well as medications, surgical space, and equipment. Rabbits must be cared for before and after their surgery, and a qualified and experienced surgeon must be available for the procedure. There is also the ethical goal of minimizing the number of animals needed to conduct research, reduction, and replacement, as described in the “3Rs” that guide the ethics of animal research [35]. Because specimens are collected after sacrifice from rabbits that are being used for other purposes, this model follows the goal of replacing research on living animals with research on inert material. By allowing researchers to obtain preliminary results prior to in vivo research, it allows for a reduction in the eventual number of animals needed to conduct that research.

### 4.2. Impact of GFs on Ex Vivo Urethral Model

The results of the GF impact using this 3D model are similar to those using a 2D model of urothelial healing. [18] IGF-1 showed the most significant wound contraction in this study, which was similar to what was found with the 2D model, which showed significant ingrowth of a single layer of urothelium when exposed to IGF-1. The findings of both the 3D and 2D models align with prior in vivo work, including that of Shinchi et al., who wrapped collagen that was impregnated with IGF-1 around a urethral catheter and showed that this decreased the strictures after urethral trauma [36]. The IGF-1 receptor is known to be prominent in the epithelium of the rat urethra [37], and has been proposed as a treatment for stress urinary incontinence [38,39].

The impact of EGF in this study was expected based on the 2D in vitro findings [18]. Prior to our 2D study, we hypothesized that EGF would have the greatest impact on wound contraction; however, we theorize that our choice to model the early epithelialization phase of urethral healing explained the lack of impact of EGF on wound contraction. EGF is known to induce growth within the urinary tracts of rats [40]; most likely, it impacts urethral healing at a later phase, and most likely, the proliferative phase that occurs between day 6 and day 10.

It is interesting to note the similarity in the results of our GF testing between the 2D model and this ex vivo 3D model. We undertook the development of the current urethral model due to the many limitations of the 2D model, most specifically because the 2D model used urothelium from a bladder source and because culturing is known to change the phenotype of urothelial cells. But given the similar results, it may be that the 2D in vitro model is a cost-effective alternative for simpler investigations of the urothelium that do not require the anatomic and cellular complexity provided by the 3D ex vivo model.

### 4.3. Other Uses for the Described Model

While we used this 3D model to evaluate the impact of GF on early urethral healing, the platform could be used for a number of different experiments. It is ideally suited for drug testing of the urethra; for example, for the treatment of urethral carcinoma or new transurethral erectile dysfunction medications [41]. It can be used to study infections ranging from bacterial to viral. It could also be used to test the mechanical properties of the urethra, such as elasticity, trauma, or shear strength, as would be of interest when developing catheters or investigating urinary incontinence [42,43,44]. This model can also be used to stabilize and evaluate other urologic tissues, such as the ureter and bladder. It can also be easily adapted to the urethra of other species, including humans, by changing the size of the 3D-printed frame.

### 4.4. Limitations

This study has a number of limitations. The first is the short time frame. This was purposely selected by the investigators, given the focus on studying early healing after a wound. Prior in vivo work shows that urethral healing progresses in phases [5,6]. Over the first 48 h, there is a single layer of urothelial cell growth, which progresses from the edges of the wound to the center and covers the surface of a urethral wound. This is the phase that is targeted to provide early epithelialization, which is thought to be the key difference between healthy healing and the fibrosis that causes urethral strictures due to reducing urinary extravasation and its resulting inflammation [8,9]. Later time points of healing involve a complex response, including leukocyte migration and collagen deposition that we do not expect can be successfully mimicked in vitro [5]. Because our clinical goal is to investigate and ultimately support early epithelialization, we elected to end the experiments after 96 h, as the “wound” model developed is not expected to mimic later time points of healing. If this model is used for experiments looking into other conditions where the physiologic response is not as important, such as drug, infection, or mechanical testing, longer time frames could be used. Penile tissues, including the urethra, are known to show high viability for 7 days in explant models [20]. We also elected to use a mechanical injury with a scalpel. This more closely mimics the injury from a surgery or trauma, which is the injury of clinical interest to our laboratory. However, urethral injuries and strictures can come from heat, such as with cautery, or from chronic inflammation, such as from sexually transmitted infections. To study other urethral diseases, a different approach could be used to create the wound.

Another limitation is the use of the distal rabbit urethra for this study, which is known as the “pars cavernosa urethrae” in rabbits but is analogous to the penile urethra in humans [27]. We elected to use the penile urethra, primarily because of the ease of harvesting it from rabbits that were otherwise being used for other purposes. It would be possible to continue a dissection into the pelvis to remove the proximal urethra. However, we found that the delicate urethral tissue tended to tear without support from the corpora cavernosa. Given the lack of surrounding cavernosum at the bulbar and prostatic urethra, these more proximal urethral locations may not be suited for the described technique. Urethral strictures are found in both the penile and bulbar urethra. Strictures in the penile urethra are more typically due to surgery or catheterization, while strictures in the bulbar urethra are typically due to iatrogenic causes [4]. The anatomy and cellular makeup of the penile urethra and bulbar urethra are different [28]; how this impacts urethra healing is not currently known. This model may be more suited to investigate healing for penile urethral strictures, or other conditions that impact the penile urethra specifically, such as catheterization trauma or lichen sclerosis.

We were not able to develop a standard or reproducible size for the wounds. Unfortunately, a punch would cut through the urethra and the skin behind, which would no longer provide a clinically relevant model of urethral injury in the absence of epidermal injury. We considered purchasing a shallow-depth laser, but then worried that this would place the model out of reach for labs that do not have this expensive piece of equipment. Ultimately, we elected to accept that we would create wounds of varying sizes and accounted for this lack of wound size standardization in how the data were recorded, analyzed, and presented. We elected to use blinded human reviewers after finding that automated wound area calculations using Image-J were not reliable due to the variation in the initial wound size. We elected to present the data as a % change from the initial area so that differences in initial wound size would be accounted for. We suspect that this increased the variability in our results, and therefore increased the number of data points required to reach statistical significance.

We elected to use commercially available cell media for this study, in part because it was similar to that used in the explant penile model [20] and, in part, to provide a direct comparison with our prior published 2D work [18]. However, a more physiologic environment would include urine, which is also present when the urethra is healing. While urine does not provide the nutrients needed to support explanted tissue, this does mean that the other components of urine that are typically present in vivo are not in this 3D model. The impact of urine on urethral healing is mixed. Urinary extravasation is thought to increase inflammation [9,45], increasing stricture formation. This is, in part, why we hypothesize that early urethral epithelialization will reduce stricture formation. However, urine also contains GFs and stem cells, which may improve healing. While adding urine to the explant media would have been more physiologic, it would also have made controlling the concentration of GF difficult since GFs are present in urine at varying concentrations [46,47]. Therefore, we felt that excluding urine is an appropriate trade-off to allow for control over the concentration and types of GFs that the specimen is exposed to.

There are some final limitations that are not easy to overcome. First, the model is flat and not tubular. While we found this to be a significant benefit in allowing us to use light microscopy for specimens, it may make this model less applicable for certain experiments. Finally, there is a lack of blood supply or immunological response in this ex vivo model. Ultimately, studies on these more complex systemic aspects will still require in vivo testing.

There are two directions for future work in this area. From a basic science perspective, we will be using this model to evaluate the early cellular responses that drive the differences in outcomes when the urethra is exposed to different GFs after injury. From a clinical perspective, our lab continues to work to develop technology that is capable of delivering GFs to the human urethra. The 3D model described here will assist us as we test these interventions to understand their impact on early urethral healing prior to in vivo and long-term animal testing.

## 5. Conclusions

This study demonstrates the feasibility of a simple and clinically relevant 3D model of the urethra using explanted rabbit tissue that was designed to investigate early urethral healing. It can show variable responses in wound contraction when exposed to different GFs over 96 h; the technique can also be used to study other aspects of the urethra, including drug delivery or mechanical properties. Our new model should increase the ability of researchers to study this important but often-overlooked area of the body.

## Data Availability

The data presented in this study are available on request from the corresponding author.

## Supplementary Materials

The following supporting information can be downloaded at: https://www.mdpi.com/article/10.3390/mps8040096/s1, Figure S1: Representative images of cell viability staining at 96 hours of no GF Control (A) and EGF 100ng/mL (B) at 10x magnification. The difficulties in obtaining high resolution images due to varying tissue depth can be seen in B, where a portion of the image on the left is out of focus; Table S1: Unnormalized mean, median, range and standard deviation for each wound.

## Abbreviations

The following abbreviations are used in this manuscript:

GF	Growth factor
EGF	Epidermal growth factor
FGF-basic	Fibroblast growth factor-basic
IGF-1	Insulin-like growth factor-1
PDGF	Platelet-derived growth factor
TGF-β1	Transforming growth factor beta
VEGF	Vascular endothelial growth factor
