# Supplementary material for: Development of an Ex Vivo Platform to Model Urethral Healing
Source: Methods Protoc. 2025 Aug 15;8(4):96. doi: 10.3390/mps8040096 (PMC12388648; doi:10.3390/mps8040096)
Supplement: Supplementary file 1 [file mps-08-00096-s001.zip › mps-3733029-supplementary.pdf]

Supplementary Information

Table S1. Unnormalized mean, median, range and standard deviation for each wound.

	Time 0				Time 24				Time 48				Time 72				Time 98			
	Mean	Median	Range	SD	Mean	Median	Range	SD	Mean	Median	Range	SD	Mean	Median	Range	SD	Mean	Median	Range	SD
**GF-Containing Control Media**																				
Wound 1	4.07	3.90	3.69 - 4.61	0.48	4.00	4.00	3.76 - 4.25	0.25	3.64	3.67	3.24 - 4	0.38	3.40	3.38	3.21 - 3.6	0.20	3.54	3.57	3.36 - 3.7	0.17
Wound 2	1.54	1.53	1.25 - 1.85	0.30	1.72	1.80	1.51 - 1.87	0.19	1.45	1.50	1.25 - 1.6	0.18	1.36	1.39	1.08 - 1.6	0.26	1.32	1.30	1.12 - 1.55	0.21
Wound 3	8.42	8.66	2.96 - 13.64	5.35	8.93	7.73	2.11 - 16.96	7.50	8.16	7.57	1.97 - 14.92	6.49	8.14	8.04	2.87 - 13.53	5.33	9.00	8.86	2.32 - 15.82	6.75
Wound 4	4.76	5.01	3.1 - 6.17	1.55	5.41	4.88	3.57 - 7.77	2.15	5.14	4.36	4.33 - 6.73	1.38	4.19	4.36	2.83 - 5.39	1.29	5.03	4.78	3.24 - 7.06	1.92
**GF-Free Control Media**																				
Wound 1	6.64	5.16	4.75 - 10.02	2.93	7.14	6.16	5.75 - 9.51	2.06	7.56	8.05	4.8 - 9.84	2.56	6.97	6.18	5.07 - 9.66	2.40	6.87	6.10	5.58 - 8.92	1.80
Wound 2	9.94	6.92	5.42 - 17.47	6.56	10.50	7.86	7.24 - 16.38	5.11	9.73	6.66	5.93 - 16.6	5.96	9.38	6.40	5.88 - 15.85	5.61	8.89	5.69	5.22 - 15.76	5.95
Wound 3	14.50	10.94	10.14 - 22.42	6.87	16.92	13.94	13.19 - 23.64	5.83	15.22	11.53	11.53 - 22.6	6.39	15.43	12.59	11.53 - 22.16	5.86	17.21	14.41	12.35 - 24.87	6.71
Wound 4	16.25	15.83	14.21 - 18.71	2.28	16.03	14.41	13.68 - 20.01	3.46	16.03	15.09	14.05 - 18.95	2.58	16.21	14.39	14.29 - 19.97	3.25	16.60	14.31	13.98 - 21.51	4.25
**IGF 100 ng/mL**																				
Wound 1	10.04	9.71	9.02 - 11.38	1.21	9.51	9.25	8.62 - 10.66	1.05	9.00	8.73	8.59 - 9.69	0.60	9.16	8.69	8.69 - 10.1	0.81	8.99	8.89	8.23 - 9.86	0.82
Wound 2	18.29	15.84	12.72 - 26.33	7.13	19.49	18.30	15.62 - 24.54	4.57	18.11	17.94	12.12 - 24.27	6.08	17.85	16.12	13.9 - 23.54	5.05	17.00	15.08	13.32 - 22.61	4.94
Wound 3	9.88	9.84	9.29 - 10.52	0.62	9.50	10.01	7.09 - 11.4	2.20	10.27	9.53	9.33 - 11.94	1.45	9.39	9.52	8.96 - 9.7	0.38	10.48	10.10	9.82 - 11.53	0.92
Wound 4	6.80	6.25	6.1 - 8.06	1.09	6.04	6.35	5.39 - 6.37	0.56	5.41	5.16	5.11 - 5.95	0.47	5.27	5.72	4.1 - 5.98	1.02	5.59	5.68	4.9 - 6.19	0.65
**IGF 10 ng/mL**																				
Wound 1	6.22	6.51	5.34 - 6.8	0.77	6.29	6.46	5.43 - 6.97	0.78	6.54	6.62	6.27 - 6.73	0.24	6.08	6.33	5.23 - 6.69	0.76	6.51	6.43	6.19 - 6.92	0.37
Wound 2	3.68	2.81	2.58 - 5.66	1.71	3.92	2.64	1.81 - 7.31	2.96	5.07	4.72	3.86 - 6.61	1.41	4.00	2.69	2.47 - 6.85	2.47	4.49	3.54	2.94 - 6.99	2.18
Wound 3	3.32	3.35	3.19 - 3.43	0.12	3.40	3.28	3.25 - 3.67	0.23	3.15	3.01	2.68 - 3.76	0.55	3.17	2.91	2.64 - 3.97	0.70	2.67	2.55	2.5 - 2.97	0.26
Wound 4	2.52	2.52	2.45 - 2.58	0.07	2.75	2.71	2.64 - 2.92	0.15	2.41	2.31	2.31 - 2.62	0.18	2.81	2.91	2.49 - 3.04	0.29	2.55	2.53	2.47 - 2.66	0.10
**EGF 100 ng/mL**																				
Wound 1	7.69	5.01	3.36 - 14.7	6.13	8.74	4.70	3.22 - 18.3	8.31	9.94	6.76	2.85 - 20.2	9.10	8.36	5.82	3.15 - 16.1	6.84	9.10	7.80	2.71 - 16.8	7.13
Wound 2	21.85	21.50	18.06 - 25.98	3.97	19.77	20.00	14.43 - 24.89	5.23	22.30	22.77	19.7 - 24.43	2.40	19.08	18.40	15.73 - 23.11	3.73	19.05	20.60	14.17 - 22.38	4.32
Wound 3	4.42	3.70	2.61 - 6.94	2.25	3.81	3.24	2.18 - 6.01	1.98	4.87	4.51	3.13 - 6.99	1.96	4.51	3.91	2.69 - 6.92	2.18	4.62	4.61	3.3 - 5.94	1.32
Wound 4	6.44	5.28	1.21 - 12.83	5.90	5.42	1.78	0.06 - 14.41	7.83	5.76	3.16	1.33 - 12.8	6.16	6.77	4.84	1.23 - 14.23	6.71	6.92	5.14	1.29 - 14.32	6.69
**EGF 10 ng/mL**																				
Wound 1	5.47	5.71	4.46 - 6.02	0.70	5.98	6.04	5.16 - 6.66	0.68	5.98	6.33	4.37 - 6.89	1.18	5.38	5.52	4.3 - 6.19	0.94	5.33	5.27	3.87 - 6.94	1.54
Wound 2	2.07	2.02	1.47 - 2.76	0.55	2.39	2.22	1.82 - 3.33	0.68	1.94	1.83	1.03 - 3.05	0.85	1.93	1.78	1.17 - 3	0.78	2.15	2.04	0.56 - 3.93	1.40
Wound 3	18.96	14.16	8.35 - 34.36	13.65	19.43	12.74	9.87 - 35.68	14.15	21.40	13.97	8.82 - 41.42	17.52	18.63	11.99	7.64 - 36.25	15.41	19.20	14.26	3.74 - 39.6	18.43
Wound 4	11.08	10.07	1.43 - 21.76	10.20	11.36	8.50	2.75 - 22.82	10.34	14.94	10.07	3.02 - 31.72	14.96	12.20	8.66	2.89 - 25.04	11.49	13.87	8.08	6.58 - 26.96	11.36

**FGF 100 ng/mL**																				
Wound 1	12.68	10.72	9.01 - 18.31	4.95	12.69	11.43	7.81 - 18.84	5.62	13.29	13.29	11.07 - 15.52	3.15	11.39	7.62	6.58 - 19.98	7.46	11.63	9.26	7.29 - 18.35	5.90
Wound 2	11.44	11.46	8.51 - 14.34	2.91	12.88	12.26	9.98 - 16.4	3.26	12.79	12.79	12.19 - 13.39	0.85	13.39	14.74	7.68 - 17.77	5.18	13.68	12.73	12.54 - 15.77	1.81
Wound 3	12.70	12.74	11.38 - 13.98	1.30	11.65	11.39	11.38 - 12.19	0.47	11.21	11.28	10.74 - 11.6	0.43	12.76	11.66	10.83 - 15.78	2.65	13.44	11.75	11.67 - 16.9	3.00
Wound 4	14.82	13.30	11.81 - 19.36	4.00	14.63	12.95	12.48 - 18.47	3.33	14.33	13.33	11.75 - 17.92	3.20	15.27	13.26	12.68 - 19.88	4.00	14.82	12.31	12.06 - 20.09	4.56
**FGF 10 ng/mL**																				
Wound 1	6.01	5.71	3.74 - 8.9	2.13	5.44	5.00	4.7 - 7.07	1.10	5.70	5.75	4.31 - 6.99	1.18	5.54	5.06	4.4 - 7.63	1.45	5.64	5.33	4.73 - 7.17	1.10
Wound 2	6.80	6.59	1.6 - 12.45	4.44	7.50	7.33	4.74 - 10.61	2.42	6.30	5.59	5.06 - 8.95	1.79	7.73	6.45	6.16 - 11.86	2.76	7.73	7.07	5.39 - 11.39	2.57
Wound 3	8.65	7.88	7.2 - 10.88	1.96	8.16	7.77	6.76 - 9.96	1.64	9.22	8.78	8.29 - 10.59	1.21	9.29	8.56	8.29 - 11.03	1.51	10.42	10.70	9.26 - 11.31	1.05
Wound 4	4.05	3.90	3.16 - 5.1	0.98	4.42	2.77	2.69 - 7.78	2.91	6.96	6.26	3.58 - 11.03	3.78	5.09	5.85	3.15 - 6.27	1.69	3.92	4.26	2.97 - 4.53	0.84
**PDGF 100 ng/mL**																				
Wound 1	11.31	11.29	11.12 - 11.53	0.20	11.61	11.84	11.1 - 11.87	0.44	10.49	10.51	10.39 - 10.58	0.10	10.48	10.27	10.11 - 11.05	0.50	10.28	10.23	9.82 - 10.78	0.48
Wound 2	7.52	7.82	6.82 - 7.91	0.61	7.99	7.86	7.69 - 8.43	0.39	6.64	6.66	6.56 - 6.68	0.07	6.11	6.12	5.71 - 6.5	0.40	6.44	6.34	6.2 - 6.8	0.31
Wound 3	4.40	4.27	3.79 - 5.25	0.68	6.28	6.40	5.53 - 6.78	0.53	4.93	5.05	3.63 - 5.98	0.99	4.02	4.04	3.75 - 4.26	0.25	3.80	3.68	3.37 - 4.46	0.48
Wound 4	4.02	2.95	2.33 - 7.86	2.58	4.74	4.43	3.05 - 7.06	1.85	4.91	4.41	3.01 - 7.82	2.24	3.73	3.13	2.88 - 5.79	1.38	4.08	3.09	2.35 - 7.79	2.52
**PDGF 10 ng/mL**																				
Wound 1	18.44	18.85	16.64 - 19.42	1.25	19.86	19.71	18.55 - 21.47	1.21	19.11	18.91	18 - 20.61	1.30	20.19	20.29	19.13 - 21.03	0.88	19.70	19.43	19.19 - 20.74	0.71
Wound 2	18.95	20.14	14.23 - 21.29	3.19	19.59	19.13	16.77 - 23.32	2.98	18.28	19.55	12.24 - 21.79	4.25	22.51	22.42	21.37 - 23.84	1.31	22.19	22.38	20.89 - 23.11	1.02
Wound 3	10.07	9.89	9.12 - 11.22	1.06	10.34	10.93	8.62 - 11.46	1.51	11.65	11.47	11.44 - 12.05	0.34	10.62	11.35	7.62 - 12.89	2.71	10.37	10.52	7.7 - 12.91	2.61
Wound 4	9.94	11.37	7.05 - 11.38	2.50	4.12	4.20	2.86 - 5.23	1.04	10.05	9.43	9.11 - 11.63	1.37	9.66	11.04	6.4 - 11.55	2.84	2.56	2.57	1.42 - 3.69	1.13
**TGF 100 ng/mL**																				
Wound 1	7.12	7.19	6.08 - 8.08	1.00	8.62	8.11	5.83 - 11.93	3.08	8.97	7.18	7 - 12.74	3.26	9.49	8.51	7.6 - 12.37	2.53	8.64	8.06	5.83 - 12.02	3.13
Wound 2	10.66	10.66	4.03 - 17.28	9.37	10.99	10.99	3.33 - 18.64	10.82	14.22	14.22	3.94 - 24.51	14.54	14.65	14.65	4.67 - 24.63	14.11	13.00	13.00	5.2 - 20.79	11.03
Wound 3	1.82	1.79	1.73 - 1.96	0.10	2.53	2.28	2.2 - 3.37	0.56	2.30	2.26	2.08 - 2.59	0.22	2.20	2.17	1.85 - 2.61	0.38	2.23	1.96	1.87 - 3.15	0.61
Wound 4	4.27	4.32	3.8 - 4.62	0.34	4.12	4.20	2.86 - 5.23	1.04	4.55	4.47	4.24 - 5.03	0.38	4.33	4.43	3.75 - 4.73	0.42	4.61	4.57	4.34 - 4.95	0.26
**TGF 10 ng/mL**																				
Wound 1	2.88	2.92	2.1 - 3.59	0.61	2.29	2.33	1.71 - 2.78	0.44	2.81	2.89	2.02 - 3.42	0.63	2.38	2.32	2.17 - 2.71	0.25	2.99	3.02	2.56 - 3.37	0.34
Wound 2	7.87	8.93	2.39 - 11.25	3.82	8.69	8.27	7.65 - 10.56	1.37	9.77	9.23	8.11 - 12.53	1.93	8.85	9.11	6.81 - 10.38	1.51	9.49	9.18	7.34 - 12.25	2.04
Wound 3	9.42	9.31	8.12 - 10.85	1.37	7.77	8.28	5.96 - 9.05	1.61	9.40	9.74	7.96 - 10.48	1.29	7.85	8.15	6.74 - 8.66	0.99	8.70	8.86	8.12 - 9.12	0.52
Wound 4	3.12	3.09	2.33 - 3.96	0.82	2.98	3.61	1.71 - 3.62	1.10	3.43	3.90	1.32 - 5.07	1.92	2.91	3.42	1.72 - 3.6	1.04	2.56	2.57	1.42 - 3.69	1.13
**VEGF 100 ng/mL**																				
Wound 1	8.11	7.81	7.65 - 8.87	0.66	8.24	8.47	6.77 - 9.48	1.37	7.76	6.92	6.5 - 9.85	1.82	8.08	8.23	7.63 - 8.39	0.40	8.27	8.83	6.99 - 8.99	1.11
Wound 2	10.19	10.19	8.28 - 12.1	2.70	11.92	11.92	10.58 - 13.27	1.90	12.86	12.86	12.37 - 13.35	0.69	12.00	12.00	11.97 - 12.03	0.04	12.34	12.34	12.22 - 12.46	0.17
Wound 3	12.78	11.37	9.58 - 17.39	4.09	13.95	14.70	10.27 - 16.88	3.37	12.96	16.69	4.04 - 18.15	7.76	17.07	17.92	14.51 - 18.78	2.26	15.24	14.77	9.86 - 21.1	5.63
Wound 4	3.07	2.99	2.74 - 3.48	0.38	3.64	3.38	3.38 - 4.18	0.46	7.55	4.56	3.77 - 14.32	5.88	4.07	4.20	3.82 - 4.2	0.22	4.67	4.71	4.51 - 4.8	0.15
**VEGF 10 ng/mL**																				

Wound 1	12.41	11.79	9.48 - 15.97	3.29	14.39	14.19	13.09 - 15.9	1.42	14.54	14.47	13.67 - 15.49	0.91	13.41	14.58	9.98 - 15.67	3.02	15.27	14.90	14.87 - 16.04	0.67
Wound 2	3.62	3.46	3.08 - 4.31	0.63	3.26	3.28	2.24 - 4.26	1.01	3.19	2.89	2.8 - 3.87	0.59	2.67	2.20	1.88 - 3.93	1.10	3.54	3.31	2.86 - 4.44	0.82
Wound 3	3.84	3.87	3.2 - 4.47	0.63	3.56	3.66	3.12 - 3.89	0.40	4.48	4.53	3.69 - 5.24	0.78	3.80	4.31	2.66 - 4.41	0.98	4.32	4.64	3.33 - 4.98	0.87
Wound 4	8.17	8.08	7.93 - 8.5	0.29	6.88	7.67	4.85 - 8.13	1.78	8.12	8.75	6.83 - 8.79	1.12	7.13	7.74	5.69 - 7.96	1.25	7.16	7.91	4.82 - 8.73	2.06

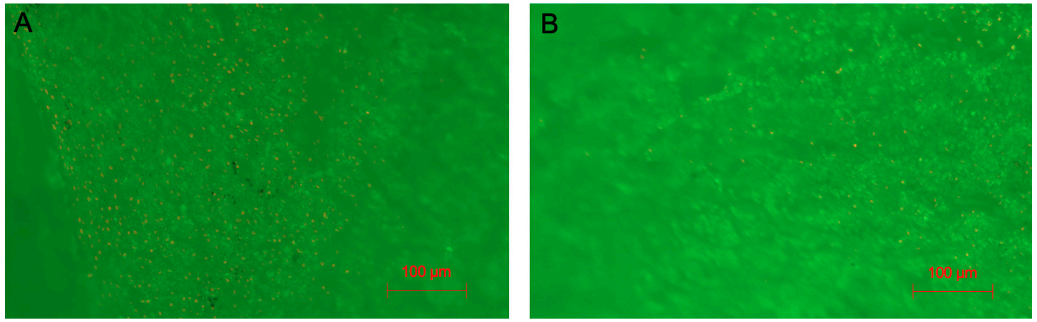

Figure S1. Representative images of cell viability staining at 96 hours of no GF Control (A) and EGF 100ng/mL (B) at 10x magnification. The difficulties in obtaining high resolution images due to varying tissue depth can be seen in B, where a portion of the image on the left is out of focus.
